# Supplementary material for: Association between low muscle mass and metabolic syndrome in elderly Japanese women
Source: PLoS One. 2020 Dec 3;15(12):e0243242. doi: 10.1371/journal.pone.0243242 (PMC7714206; doi:10.1371/journal.pone.0243242)
Supplement: Attachment — Submitted filename: Response_to_Reviewers.docx [file pone.0243242.s001.docx]

**Response to Reviewers**

Reviewer 1

1-1. Authors described participants of this study as healthy female subjects. I could not understand the reason why healthy subjects received medical check-ups routinely at clinic. According to the description, around 40% of subjects were received antihypertensive medications. I think it is rather natural if most of subjects of this age received some medical treatment for the chronic illness than they were completely healthy..

RESPONSE: Thank you for your comments. The definition of healthy has been clarified in the manuscript (see lines 87 – 90).

1-2. Why did the authors select only female subjects? Was their clinic specialized only for female?

RESPONSE: Thank you. The rationale for focusing on older women is explained in more detail in the manuscript text. Women represent a large majority of older adults receiving treatment for functional impairments in the Japanese population and are especially vulnerable to negative health impacts associated with metabolic syndrome (lines 47 – 60).

1-3. Almost 80% of the subjects had hypertension. So, when the authors referred to the relations between skeletal mass/visceral fat mass and metabolic syndrome, the existence of impaired fasting glucose and/or dyslipidemia affected the results. The authors should add the detailed information about the relation between each component of metabolic syndrome and skeletal mass/visceral fat mass.

RESPONSE: The rate of hypertension in this sample is similar to that of the population of older adults in Japan as a whole (please see lines 165 – 166).

1-4. The authors demonstrated SMI in Figure 1, however, they demonstrated ASM in figure2. Also, they analyzed multiple regression analysis by ASM in Table 2. How did they use them differently? Is SMI better clinical marker as it is corrected by height?

RESPONSE: SMI was used in bivariate analyses because it controls for height. The multivariate analyses use other factors to control for body size, so uncontrolled ASM is used in those analyses instead. This is now explained in the methods section (see lines 135 – 141).

Reviewer 2

2-1. Poor choice of MetS as a surrogate for CVD and risk:
•In this cohort, MetS was not very representative of CVD as noted by the fact that the most common MetS was SBP in 77.8% Surprisingly, of this number only 41.9% required medication/treatment.
•Further only 2.2% of patients had a history of CAD.
•Dyslipidemia and dysglycemia was much less common.
• Thus, MetS as defined by this study, was an outcome not as representative of CAD/CVR, and use of a subclinical marker such as Coronary calcification CT, carotid US would have been more representative of CVR.

RESPONSE: Thank you for your comments. The scope of the manuscript has been re-framed in the present version to focus specifically on metabolic syndrome, rather than on cardiovascular health overall.

2-2. Poor generalizability:
•Recruitment of only women, and in one clinic in Japan means the results cannot be generalized to populations in the west such as US, where MetS definitions would be more likely to show higher markers of obesity
dyslipidemia, and dysglycemia.

RESPONSE: The scope of the manuscript has been re-framed to focus more narrowly on Japanese women specifically, rather than older adults in general.

2-3. Questionable originality:
•The importance of skeletal muscle mass and CVR in older adults has been evaluated in many other cohorts, including KNHANES, HealthABC, NHANES and while inclusion of VFA to more accurately quantify adiposity is laudable it does not represent a significant enough advance

RESPONSE: We have modified the discussion section to highlight the contributions of this paper (see lines 278 – 286). Additionally, we would like to note that the PLoS ONE criteria for publication state that perceived significance or impact of the results do not constitute a criterion for acceptance or rejection of a manuscript.

2-4. Cross sectional study:
•Relationships noted are interesting but should have been confirmed over time
•Cross sectional nature adds to the limited scientific utility of the study

RESPONSE: We have modified the discussion section to highlight the contributions of this paper (see lines 278 – 286). Additionally, we would like to note that the PLoS ONE criteria for publication state that perceived significance or impact of the results do not constitute a criterion for acceptance or rejection of a manuscript.

2-5. Poorly structured abstract, with no statement of results.

RESPONSE: The abstract has been updated to include additional details regarding results. Regarding structure, the PLoS ONE style guide indicates that both unstructured and structured abstract formats are accepted. We are prepared to use a structured format if the editor requests us to do so.

2-6. Why was SMI interchanged with ASM in Figure 1?
•What is the biological import of the positive association between SMI and HOMAIR?
•This association contradicts the negative association between ASM and CVR. It needs to be reconciled.

RESPONSE: SMI was used in bivariate analyses because it controls for height. The multivariate analyses use other factors to control for body size, so uncontrolled ASM is used in those analyses instead. This is now explained in the methods section (see lines 135 – 141).
